# MicroRNA-148a-3p inhibits progression of hepatocelluar carcimoma by repressing SMAD2 expression in an Ago2 dependent manner

**DOI:** 10.1186/s13046-020-01649-0

**Published:** 2020-08-04

**Authors:** Zhao Huang, Jingyuan Wen, Jingjing Yu, Jingyu Liao, Sha Liu, Ning Cai, Huifang Liang, Xiaoping Chen, Zeyang Ding, Bixiang Zhang

**Affiliations:** 1 Hepatic Surgery Center, Tongji Hospital, Tongji Medical College, Huazhong University of Science and Technology, 1095 Jiefang Avenue, Wuhan, 430030 China; 2 Clinical Medical Research Center of Hepatic Surgery at Hubei Province, Wuhan, China; 3 Key Laboratory of Organ Transplantation, Ministry of Education, Wuhan, China; 4 Key Laboratory of Organ Transplantation, National Health Commission, Wuhan, China; 5 Key Laboratory of Organ Transplantation, Chinese Academy of Medical Science, Wuhan, China

**Keywords:** HCC, SMAD2, miR-148a, Argonaute 2, TGF-β

## Abstract

**Background:**

Hepatocellular carcinoma (HCC) is one of the most prevalent common cancer worldwide with high mortality. Transforming growth factor-β (TGF-β) signaling pathway was reported dysregulated during liver cancer formation and progression. As a key component of TGF-β signaling, the role of SMAD2 and its regulatory mechanisms in HCC remain unclear.

**Methods:**

SMAD2 expression in paired HCC specimens were determined by western blot and immunohistochemistry (IHC). quantitative real-time PCR (qRT-PCR) was used to measure mRNA and microRNA (miRNA) expression level. Cell migration, invasion and proliferation ability were evaluated by transwell, CCK8 and EdU assay. In silico websites were used to manifest overall survival rates of HCC patients or to predict miRNAs targeting SMAD2. Dual luciferase reporter assay and anti-Ago2 immunoprecipitation assay were performed to confirm the binding between SMAD2 mRNA and miRNA-148a-3p (miR-148a). Tumorigenesis and lung metastasis mouse model were used to explore the role of miR-148a in vivo. In situ hybridization (ISH) was conducted to determine the expression of miR-148a in liver tissues.

**Results:**

In this study, we found that SMAD2 was highly expressed in HCC and elevated SMAD2 expression predicted shorter overall survival (OS) time for HCC patients. SMAD2 promoted mobility and proliferation of HCC cells in vitro. We further revealed that the expression of miR-148a was negatively correlated with SMAD2 and found that miR-148a repressed SMAD2 expression by downregulating its mRNA through binding with Argonaute 2 (Ago2) in HCC. Transwell, CCK8 and animal experiments exhibited miR-148a inhibited metastasis and proliferation of HCC in vitro and in vivo. Moreover, the phenotype changes caused by miR-148a manipulation were recovered by rescuing SMAD2 expression in HCC cells. ISH assay indicated miR-148a was downregulated in HCC and low expression of miR-148a associated with more aggressive clinic features and poor prognosis.

**Conclusion:**

miR-148a was identified as a repressor of HCC progression by downregulating SMAD2 in an Ago2 dependent manner.

## Background

Liver cancer is the sixth most common cancer in incidence and the fourth leading cause of cancer-related death worldwide [1, 2]. Hepatocellular carcinoma (HCC) is the major type of liver cancer, accounting for 75–85% of primary liver cancers [3]. The main risks for HCC are hepatitis B virus (HBV), hepatitis C virus (HCV), aflatoxin contamination in food, alcohol abuse, obesity and so on [3]. Treatments for liver cancer patients contain surgical therapies, tumor ablation, transarterial therapies and systemic therapies [2]. However, the prognosis remains miserable, 5-years survival rate for liver cancer patients is 18%, only inferior to pancreatic cancer [1].

TGF-β signaling pathway has been reported playing a multifaceted role in tumor development and progression. It promotes homeostasis and suppresses tumor progression by inducing cytostasis, differentiation, apoptosis, inflammation depression and stroma-derived mitogens in normal and premalignant cells. Meanwhile, in advanced cancer cells, TGF-β initiates immune evasion, growth factor production, differentiation to invasive phenotype, metastatic dissemination and metastatic colonies establishment [4]. Eight SMAD proteins were identified participating in TGF-β signaling: the receptor-regulated SMAD (R-SMAD) (SMAD1, 2, 3, 5 and 8), the co-mediator SMAD (Co-SMAD) (SMAD4), and the inhibitory SMAD (I-SMAD) (SMAD6 and 7) [5]. As R-SMAD, SMAD2 and SMAD3 are phosphorylated by receptors of TGF-β branch, whereas SMAD1, 5 and 8 are phosphorylated by other branches of receptors such as BMP receptors [6]. Upon TGF-β stimulation, SMAD2 and SMAD3 are phosphorylated and activated by type I receptor kinase, and bind with SMAD4 to form activated SMAD complexes [5]. Regardless of the similar working mechanisms and high identity of protein structures [7], the role of SMAD2 and SMAD3 are often different, even opposite. Distinct effects of SMAD2 and SMAD3 were reported in breast cancer bone metastasis [8], pancreatic ductal adenocarcinoma cell proliferation and migration [9], HaCaT keratinocyte cell growth [10], TGF-β autoinduction in clostridium butyricum-activated dendritic cells [11] and TGF-β induced transcription [12]. Our lab has previous reported SMAD3 promoted HCC metastasis by upregulating protein tyrosine phosphatase receptor epsilon (PTPRε) expression and high expression of SMAD3 was associated with poor prognosis in HCC [13], but the impacts of SMAD2 on HCC remain largely unclear.

MicroRNAs (miRNAs) are short endogenous RNAs 19–25 nucleotides in length that post-transcriptionally regulate mRNA expression [14]. Instead of directly silencing targeted mRNA, miRNAs in human and other bilaterian animals promote mRNA decay or repress mRNA translation mainly by recruiting RNA-induced silencing complex (RISC). As the heart of RISC, Argonaute2 (Ago2) recruits TNRC6, PABPC, and deadenylase complexes, i.e., the PAN2-PAN3 complex or CCR4-NOT complex to destabilize or repress mRNA [15–17]. Numerous miRNAs have found dysregulated in HCC and involved in HCC diagnosis, prognosis and therapeutics, such as, miR-16, miR-92a and miR-500 [18–20]. Lower miR-148a expression in HCC tissues was observed than that in adjacent noncancerous hepatic tissues and miR-148a-3p expression was correlated to clinical TNM stage, metastasis, status of capsular infiltration and numbers of tumor nodes in HCC [21]. Xu et al. reported that hepatitis B virus X protein (HBx) repressed miR-148a expression and further upregulated the expression of hematopoietic pre–B cell leukemia transcription factor interacting protein (HPIP) to enhance tumorigenesis [22]. Gailhouste et al. revealed that miR-148a promoted hepatospecific phenotype of mouse fetal hepatoblasts (MFHs) and suppressed the invasiveness of transformed cells [23].

Our study revealed that SMAD2 exhibited oncogenic role in HCC and found that miR-148a was an upstream regulator of SMAD2 by decaying SMAD2 mRNA in an Ago2 dependent manner. miR-148a inhibited the mobility and proliferation of HCC cells and low expression of miR-148a in HCC was associated with shorter overall survival time. These results indicated miR-148a could be a promising diagnostic marker and therapeutic target for HCC patients.

## Methods

### Patients and tissue specimens

Human tumor and adjacent non-tumor tissues were collected from HCC patients underwent hepatectomy at the Hepatic Surgery Center, Tongji Hospital of Huazhong University of Science and Technology (HUST) (Wuhan, China). All procedures were approved by the Ethics Committee of Tongji Hospital, HUST and conducted according to the Declaration of Helsinki Principles. Prior written and informed consent was obtained from each patient.

### Cell lines and culture

HCC cell lines MHCC-97H, HCC-LM3 were obtained from Liver Cancer Institute, Zhongshan Hospital, Fudan University, Shanghai, China. Hep3B, Huh7, PLC/PRF-5 (ALEX), HLF, Bel7402 and normal liver cell line HL7702 were purchased from China Center for Type Culture Collection (CCTCC, Wuhan, China). ALEX was purchased from cell bank of Chinese Academy of Sciences (Shanghai, China) [24]. All cell lines were maintained in Dulbecco Modified Eagle Medium (DMEM) (Hyclone, UT, USA) supplemented with 10% fetal bovine serum (FBS) (Gibico) at 37 °C in a 5% CO_2_ cell incubator.

### Cell counting kit 8 assay and EdU incorporation assay

Cell Counting Kit 8 assay (Dojindo, Kumamoto, Japan) was performed according to manufactures’ protocol. Briefly, 1500 indicated cells were seeded into 96-well plates. Culture medium was changed to 100 μl 10% CCK8 solution at the indicated time and incubated in cell incubator for 2 h. Optical density (O.D.) was measure by Universal Microplate Reader ELx 800 (BIO-TEK, USA) at 450 nm wave length. For each group, the absorbance values were measured by five replicates. After extracting blank value, an average of gross O.D. values was used for data analysis.

HCC cells (4000 cells/ well) were seeded into 96-well plates and cultured overnight for EdU incorporation assay by using Cell-Light™ EdU Apollo567 In Vitro Imaging Kit (Ribobio, Guangzhou, China) according to the manufacturer’s instructions. Briefly, 100 μl 50 μM EdU solutions were added into cells and cultured for 2 h. Cells were rinsed with PBS, fixed with 4% paraformaldehyde and incubated with 0.5% TritonX-100. Then, cells were stained with 100 μl 1X Apollo solution for 30 min, nucleus were stained with 1X Hoechst33342 solution. Representative images were captured with EVOS FL auto imaging system (life technologies, USA) and positive cells were counted by Image Pro. Plus version 6.0.

### Dual luciferase assay

The sequence of 8284–8390, containing predicted binding site with miR-148a, in the 3′-untranslated region (3’UTR) of SMAD2 or 3′UTR-SMAD2-mutant was cloned into the psiCHECK™-2-vector (Promega, Madison, WI, United States). About 1 × 10^5^ cells/well were seeded into 24-well plates. After 24 h, the recombinant plasmid pSicheck-2-3′UTR-SMAD2 or pSicheck-2-3′UTR-SMAD2-mutant were co-transfected into cells with miR-148a mimic, miR-148a inhibitor or their respective negative control (nc) using Lipofectamine 3000 (Invitrogen). Forty-eight h later, total protein from cells were extracted by Passive Lysis Buffer (Promega) and the luciferase activity was determined using Dual-Luciferase® Reporter 1000 Assay System (Promega) by GloMax 20/20. Firefly luciferase values were normalized against Renilla luciferase activity, and the ratio of firefly/Renilla luciferase activity was presented.

### Anti-Ago2 RNA binding protein immuno-precipitation (RIP) assay

Rabbit anti-Ago2 IgG was purchased from Abcam (ab32381). Magna RIP™ RNA-Binding Protein Immunoprecipitation Kit (Millipore, Darmstadt, Germany) was used to enrich Ago2 binding RNA. The enriched RNA was subjected to qRT-PCR. 2^-ΔCT^ was calculated and normalized to the 2^-ΔCT^ of 10% input.

### Animal experiments

4–5 weeks old male BALB/cA-nude mice were purchased from Beijing HFK Bioscience Co. Ltd. and maintained at SPF conditions. All animal experiments were approved by the Ethics Committee of Tongji Hospital, HUST. The whole procedure was in accordance with the “Guide for the Care and Use of Laboratory Animals” (NIH publication 86–23 revised 1985). For xenograft tumor model, 1× 10^6^ indicated tumor cells were suspended in 100 μl serum free DMEM and inoculated subcutaneously into the flanks of nude mice. Thirty days later, all mice were sacrificed to compare the volume and weight of tumors. For lung metastasis model, 1 × 10^6^ cells were suspended in 100 μl serum free DMEM. Then cells were injected via the tail vein of nude mice. After 2 months of injection, mice were sacrificed and lungs were resected to calculate metastatic nodules.

### In situ hybridization (ISH) and immunohistochemistry analysis (IHC)

ISH was performed using the ISH Kit (Boster, Bio-Engineering Company, Wuhan, China). All procedures followed the manufacturer’s instructions. Samples were stained with hematoxylin, dehydrated with alcohol, washed with xylene, sealed with flavor sealing tablets. Oligo (5’Digoxin-ACAAAGTTCTGTAGTGCACTGA) was used as ISH probe for miR-148a. IHC staining was performed as described previously [24]. Primary antibody for SMAD2 (1:100, 12,570–1-AP) and Ki67 (1:100, 27,309–1-AP) were purchased from Proteintech. The representative images of ISH and IHC were captured and processed using DM2300 microscope and ScopeImage 9.0 software (Nanjing Jiangnan Novel Optics Co., Ltd., China). ISH and IHC staining scores were independently determined by 3 pathologists without prior knowledge of patient information. The overall score defined by multiplying the percentage of positive cells by the staining intensity score as described previously [24].

### In silico analysis and prediction websites

Overall survival rates of HCC patients with different SMAD2 or SMAD3 level were analyzed by GEPIA [25] and Kaplan Meier-plotter [26] websites. TargetScan [27], miRTarBase [28] and miRcode [29] websites were used to predicted miRNAs targeting SMAD2 mRNA. StarBase [30] website was used to analyzed correlations between expression of miRNAs and SMAD2 mRNA, or overall survival rate of liver hepatocellular carcinoma (LIHC) patients with different miRNAs expression.

### Statistical analyses

We used Prism 7.0 (GraphPad Software, La Jolla, CA, USA) or SPSS 13.0 (SPSS, Chicago, IL, USA) to analyze the data. Quantitative data were analyzed by Student’s *t* test (unpaired two-tailed comparison) or Pearson’s correlation test. Kaplan-Meier and log-rank analysis were applied to evaluate survival between two groups. Categorical data were analyzed by *chi-square* test. All values were presented as mean ± SEM. *P* values less than 0.05 were considered statistically significant.

**Supplementary materials and methods were provided in additional information.**

## Result

### SMAD2 and SMAD3 are upregulated in HCC specimens and their high expression predict poor prognosis

**Fig. 1:**
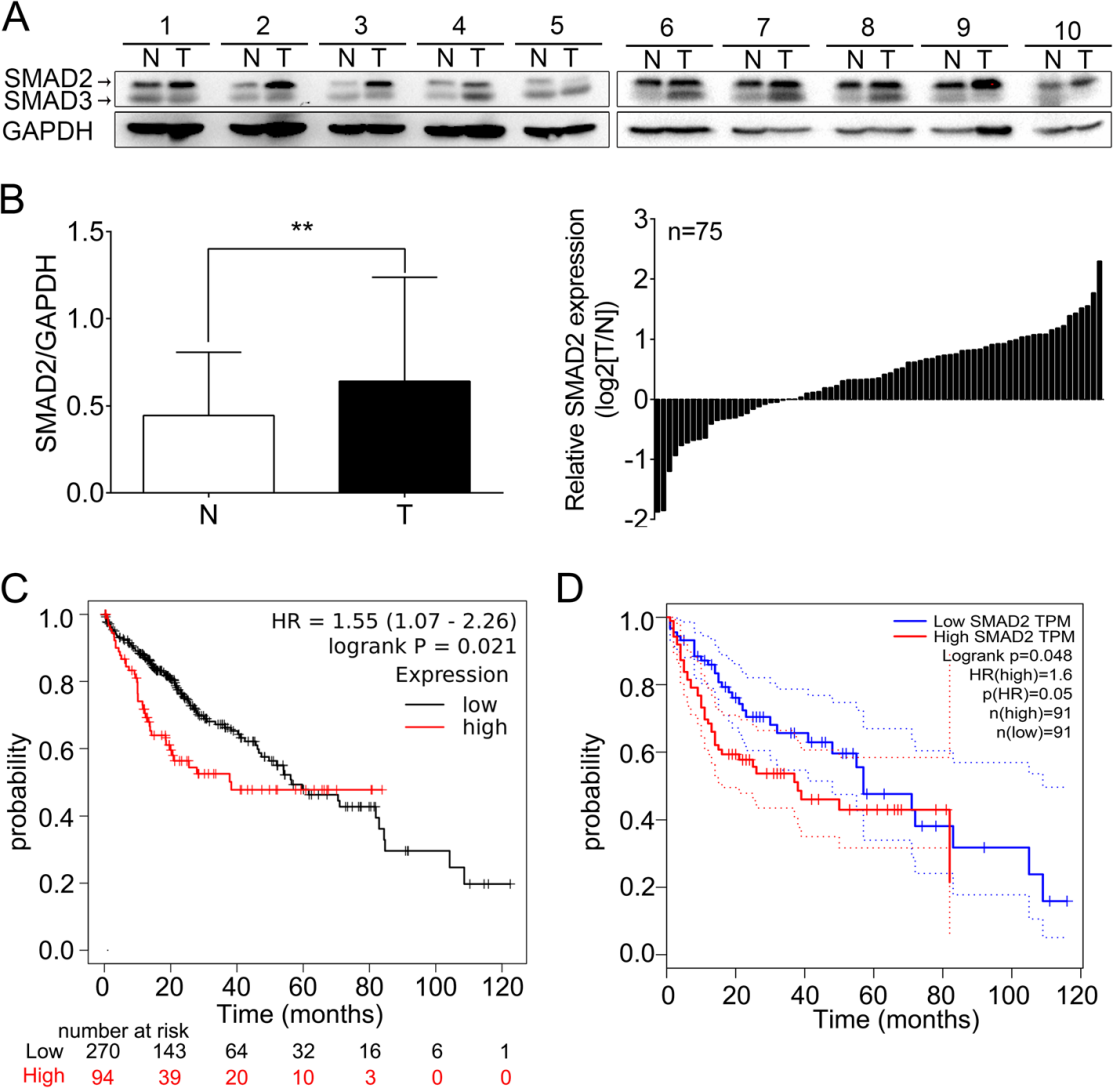
SMAD2 is upregulated in HCC specimens and high expression of SMAD2 predicted poor prognosis. **a** Representative western blot bands of SMAD2 and SMAD3 in 75 pairs HCC clinic samples. GAPDH as loading control. **b** Quantification of SMAD2 bands intensity in liver tumor and non-tumorous tissues (left panel). Relative SMAD2 expression in HCC tissues by normalizing to their respective adjacent non-cancerous liver tissues (right panel). N, non-tumorous tissues. T, tumor tissues. **c** and **d** Kaplan-Meier analysis of the correlation between SMAD2 expression and overall survival in HCC patient cohorts by Kaplan Meier-plotter (**c**) or GEPIA (**d**) website. Data are shown as Mean ± SEM. **, *p* < 0.01

We firstly examined the expression of SMAD2 and SMAD3 in 75 pairs of HCC and adjacent non-cancerous tissues by western blot (Fig. 1a and Supplementary Figure 1). The average band intensities of SMAD2 and SMAD3 were significantly higher in HCC tissues than adjacent non-cancerous tissues (Fig. 1b and Supplementary Figure 2A) and their expression levels were correlated positively in HCC samples (Supplementary Figure 2B). We then explored the relevance between SMAD2 or SMAD3 expression and patient prognosis using GEPIA and Kaplan Meier-plotter websites, higher expression of SMAD2 predicted shorter overall survival (OS) in both patient cohort (Fig. 1c and d). While elevated expression of SMAD3 was associated with poorer prognosis only in GEPIA patient cohort (Supplementary Figure 2C and 2D). Given the role of SMAD3 in HCC had been investigated in detail [13], we focused on exploring the impacts of SMAD2 in HCC.

### SMAD2 promotes migration, invasion and proliferation of HCC cells

**Fig. 2:**
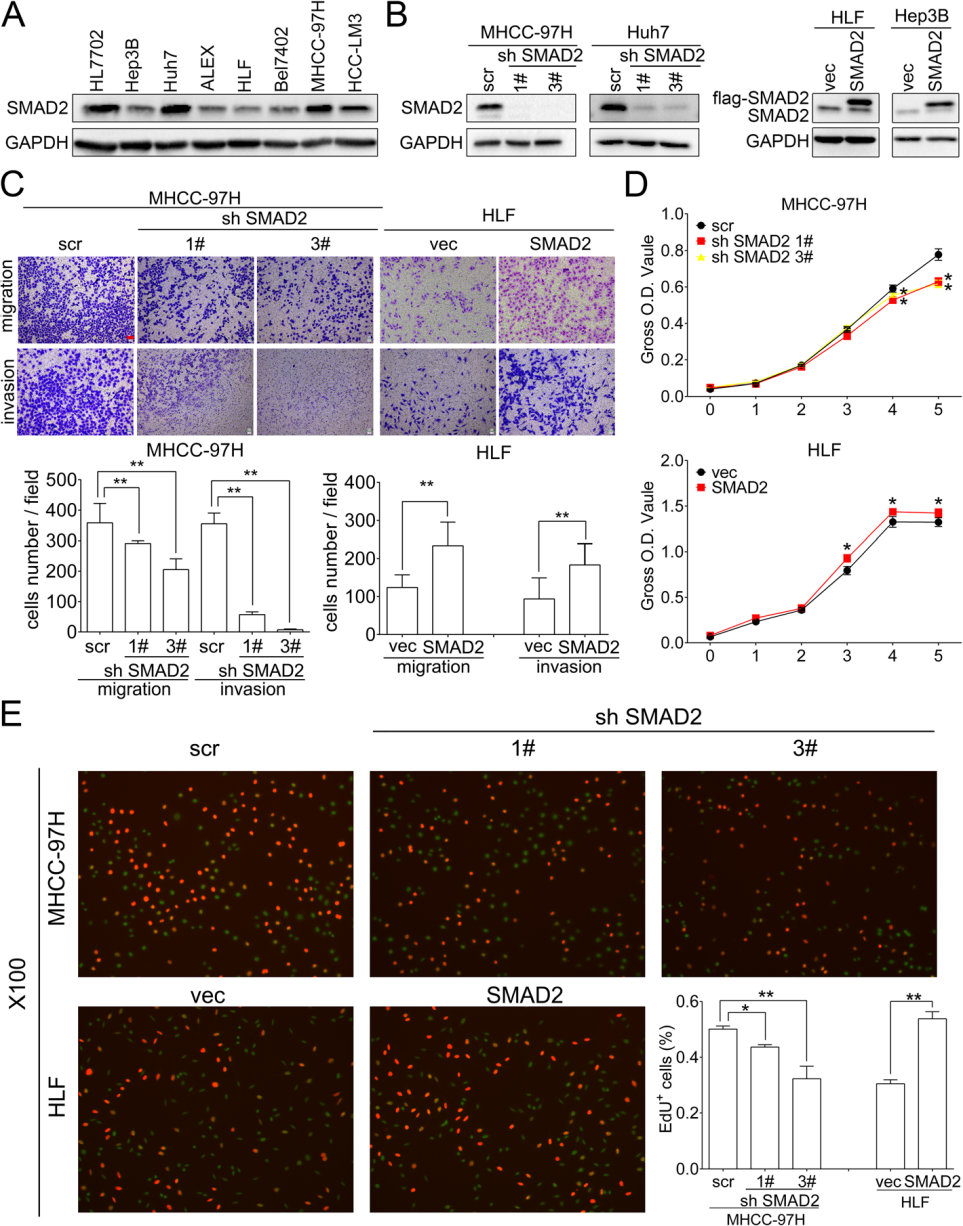
SMAD2 promotes migration, invasion and proliferation of HCC cells. **a** Western blot analysis of endogenous SMAD2 expression in HCC cell lines. GAPDH as loading control. **b** Western blot analysis of SMAD2 knocking down or overexpression efficiency in the indicated cells. GAPDH as loading control. **c** Representative images of migration and invasion assays after stably knocking down or overexpressing SMAD2 in MHCC-97H or HLF cells (upper panel). Scale bar: red bar, 25 μm. Quantification of cells migrated or invaded (lower panel). **d**, **e** CCK8 and EdU assays after stably knocking down or overexpressing SMAD2 in MHCC-97H or HLF cells. Quantification of percentages of Edu^+^ cells. Data are shown as Mean ± SEM. *, *p* < 0.05. **, *p* < 0.01

The endogenous SMAD2 expression was found relatively low in Hep3B, ALEX, HLF and Bel7402 cells, meanwhile high in Huh7, MHCC-97H and HCC-LM3 cells (Fig. 2a). We successfully knocked down SMAD2 in MHCC-97H and Huh7 cells by two independent shRNA (MHCC-97H/sh SMAD2, Huh/sh SMAD2), and overexpressed SMAD2 in HLF and Hep3B cells (HLF/SMAD2, Hep3B/SMAD2) (Fig. 2b and Supplementary Figure 3A). Transwell assay indicated that overexpression of SMAD2 promoted and knockdown of SMAD2 inhibited migration and invasion in HCC cells (Fig. 2c and Supplementary Figure 3B). CCK8 and EdU assays showed that HCC cells with higher SMAD2 expression exhibited stronger proliferation ability compared with counterpart lower SMAD2 expression cells (Fig. 2d, e, Supplementary Figure 3C and 3D).

### The mRNA of SMAD2 is targeted by miR-148a

**Fig. 3:**
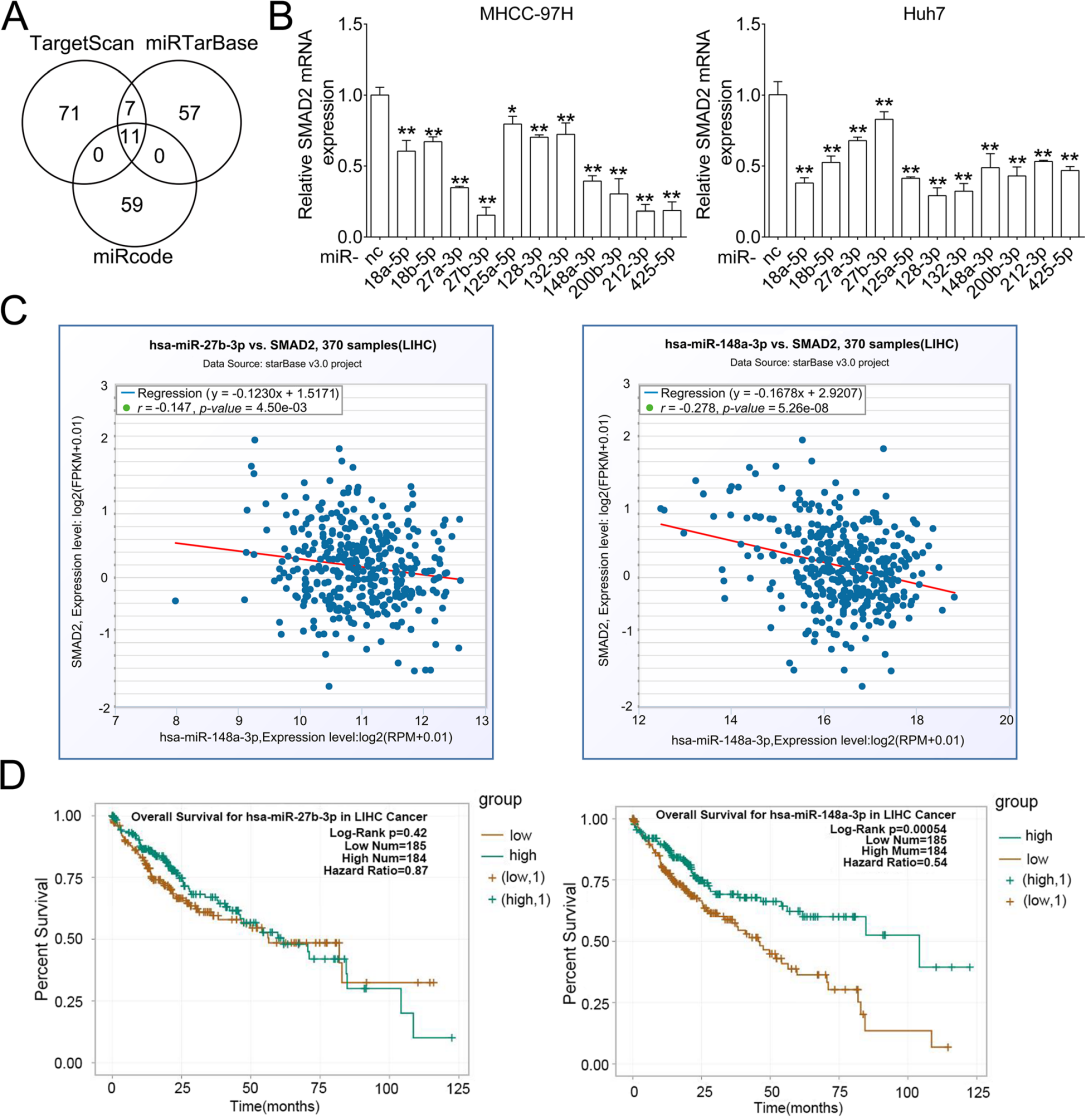
The mRNA of SMAD2 is targeted by miR148. **a** Overlap of the predicted miRNAs targeting SMAD2 mRNA from three different websites. **b** qRT-PCR analysis of SMAD2 mRNA level after transfecting the indicated miRNA mimics for 48 h in MHCC-97H and Huh7 cells. nc: negative control. Data were normalized to GAPDH and are shown as the fold change to nc. Data are shown as Mean ± SEM. **, *p* < 0.01. **c** The correlation analysis of miR-27b or miR-148a and SMAD2 expression in HCC samples by StarBase website. **d** The Kaplan-Meier analysis between miR-27b or miR-148a and overall survival in HCC cells by StarBase website. LIHC: liver hepatocellular carcinoma

To identify the candidate upstream miRNAs of SMAD2, we used in silico websites to predict miRNAs which potentially bind with SMAD2 mRNA. Eleven miRNAs were co-predicted in TargetScan, miRTarBase and miRcode websites (Fig. 3a and Supplementary Table 1). Then, we overexpressed these 11 miRNAs respectively in MHCC-97H and Huh7 cells by miRNA mimics, qRT-PCR revealed that SMAD2 mRNA level was downregulated after transfected with these miRNA mimics (Fig. 3b). Given miRNA mainly exerts its role by binding and decaying targeted mRNA [14, 17], we analyzed the mRNA expression relevance between SMAD2 and these miRNA in HCC samples by StarBase website. The mRNA level of miR-27b-3p (miR-27b) and miR-148a were found in negative correlation with SMAD2 mRNA (Fig. 3c, Supplementary Figure 4). We further analyzed the relevance between these 2 miRNAs and HCC patients’ OS. Patients with higher miR-148a expression exhibited longer OS, while miR-27b showed no significant difference (Fig. 3d). Therefore, we speculated miR-148a was a potential regulator of SMAD2 in HCC.

### MiR-148a inhibits the expression of SMAD2 by downregulating its mRNA

**Fig. 4:**
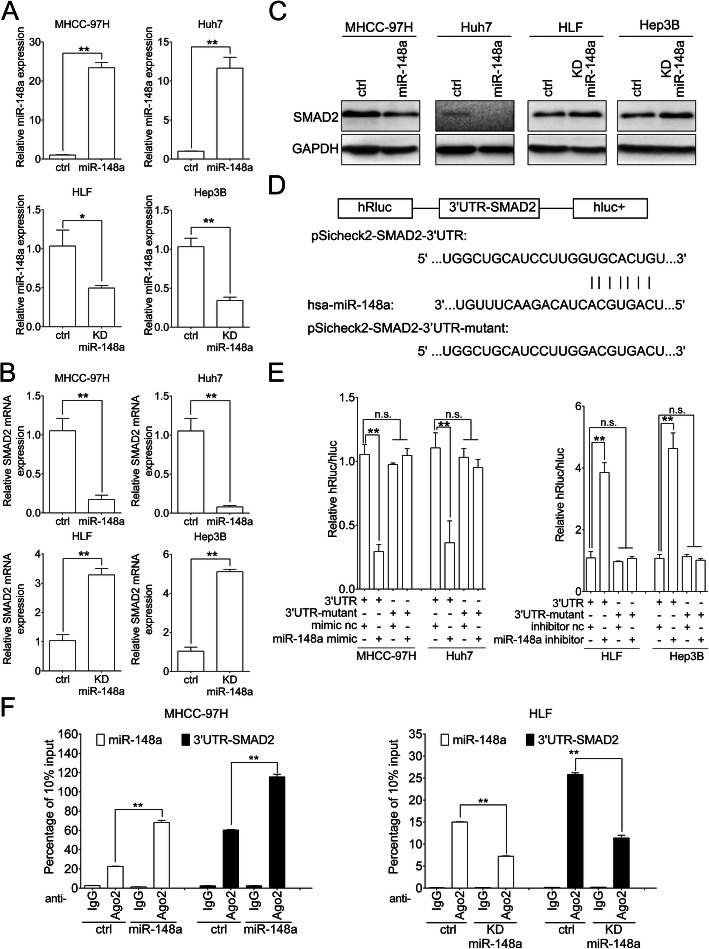
miR-148a inhibits the expression of SMAD2 by downregulating its mRNA. **a** qRT-PCR analysis of miR-148a overexpression (miR-148a) or knocking down (KD miR-148a) efficacy in the indicated cells. Ctrl, cells transfected with control lentivirus. Data were normalized to U6 and are shown as the fold change to their respective control cells. **b**, **c** qRT-PCR (**b**) and western blot (**c**) analysis of SMAD2 mRNA and protein level after stably overexpressing or knocking down miR-148a in MHCC-97H and Huh7 or HLF and Hep3B cells respectively. Data were normalized to GAPDH and are shown as the fold change to their respective control cells in (**b**). GAPDH, as loading control in (**c**). **d** Schematic image of binding site between miR-148a and SMAD2–3′-UTR. **e** The indicated cells were co-transfected with pSicheck2-SMAD2–3’UTR (pSicheck2-SMAD2–3’UTR-mutant) and miR-148a mimic (miR-148a inhibitor). Forty-eight h after transfection, cells were subjected to dual luciferase assay. (**f**) RIP assays of miR-148a and 3’UTR-SMAD2 enrichments by anti-IgG or anti-Ago2 antibodies in miR-148a overexpressing MHCC-97H cells (left panel) and miR-148a knocking down HLF cells (right panel). Data were normalized to 10% input. Data are shown as Mean ± SEM. *, *p* < 0.05. **, *p* < 0.01. n.s., no significance

We stably overexpressed miR-148a in MHCC-97H and Huh7 cells (MHCC-97H/miR-148a, Huh7/miR-148a) with high endogenous SMAD2 level and knocked down miR-148a in HLF and Hep3B cells (HLF/KD miR-148a, Hep3B/KD miR-148a-) with low endogenous SMAD2 level by lentivirus (Fig. 4a). qRT-PCR and western blot demonstrated that overexpressing miR-148a downregulated and knocking down miR-148a upregulated the SMAD2 mRNA and protein level (Fig. 4b and c). TargetScan predicted that the position 8334–8340 of SMAD2 3′ UTR was potential binding site of miR-148a. We constructed dual luciferase reporter plasmids pSicheck2-SMAD2–3’UTR and pSicheck2-SMAD2–3’UTR-mutant (Fig. 4d). Dual luciferase reporter assay showed that miR-148a mimic inhibited and miR-148a inhibitor enhanced reporter luciferase activity and the changes were abolished after mutating the binding site (Fig. 4e). Anti-Ago2 RNA immunoprecipitation (RIP) assays indicated that Ago2 enriched miR-148a and 3’UTR of SMAD2. The enrichments increased in miR-148a overexpressing MHCC-97H cells and decreased in miR-148a knocking down HLF cells (Fig. 4f).

### MiR-148a inhibits migration, invasion and proliferation ability of HCC cells in vitro

**Fig. 5:**
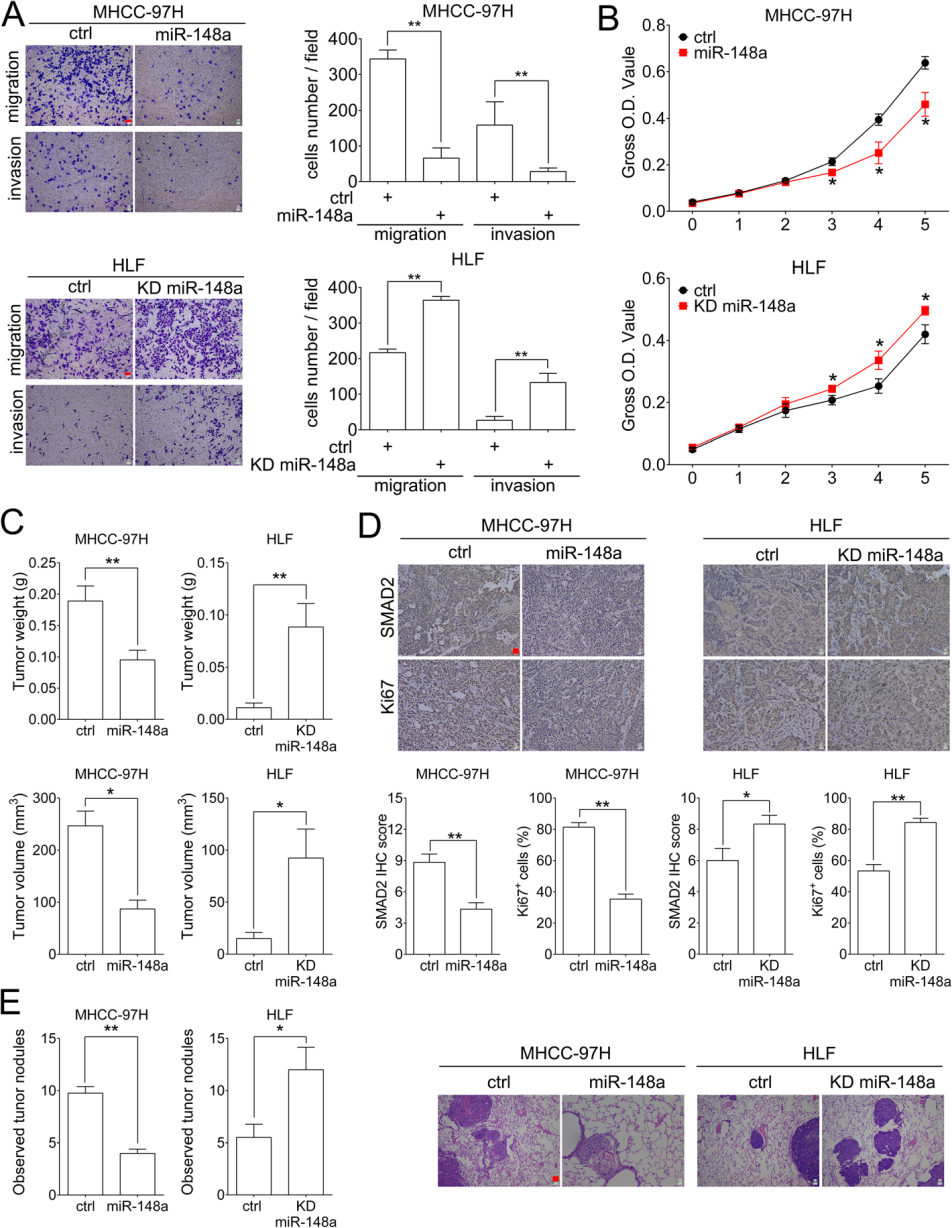
miR-148a inhibits mobility and proliferation of HCC cells in vitro and in vivo. **a** Representative images of migration and invasion assay in the miR-148a overexpressing MHCC-97H or miR-148a knocking down HLF cells. Scale bar: red bar, 25 μm. Quantification of cells migrated and invaded. **b** CCK8 assay for the indicated cells. **c** BALB/c nude mice were subcutaneous injected with the indicated cell. Quantification for tumor volume and weight of subcutaneous tumors in the indicated groups. **d** Representative images of SMAD2 and Ki67 staining of subcutaneous tumors in the indicated groups. Scale bar: red bar, 25 μm. Quantification of SMAD2 IHC scores and Ki67^+^ HCC cell percentages. **e** HCC cells were injected into nude mice by tail veins to establish HCC lung metastasis animal model. Quantification of observed metastatic tumor nodules during autopsy and metastatic niches in H&E stained slides. **f** Representative images of H&E staining for lung. Scale bar: red bar, 25 μm. Data are shown as Mean ± SEM. *, *p* < 0.05. **, *p* < 0.01

To investigated the role of miR-148a in HCC cells, we performed transwell and CCK8 assays after manipulating miR-148a expression. Overexpressing miR-148a inhibited migration and invasion in MHCC-97H and Huh7 cells, knocking down miR-148a promoted migration and invasion in HLF and Hep3B cells (Fig. 5a and Supplementary Figure 5A). CCK8 assay showed that overexpression of miR-148a inhibited and knockdown of miR-148a promoted proliferation in HCC cells (Fig. 5b and Supplementary Figure 5B).

### MiR-148a inhibits tumorigenesis and lung metastasis of HCC cells in vivo

HCC cells were injected to nude mice subcutaneously to investigated the role of miR-148a on tumorigenesis. The volume and weight of subcutaneous tumors significantly decreased after overexpressing miR-148a in MHCC-97H cells and increased after knocking down miR-148a in HLF cells (Fig. 5c and Supplementary Figure 5C). IHC staining of SMAD2 and Ki67 indicated that HCC cells with higher miR-148a expression exhibited lower SMAD2 expression and weaker proliferation abilities (Fig. 5d). We then established HCC lung metastasis animal model by injecting cancer cells via tail veins. Less metastatic nodules were observed in post-mertem lungs of mice injected with miR-148a overexpressing MHCC-97H cells compared with control cells, and H&E staining was further performed to verify the metastatic lesion formation. Similar results were obtained in mice injected with miR-148a knocking down HLF groups (Fig. 5e and Supplementary 5D). 

### The role of miR-148a in HCC cells was mediated by SMAD2 and low expression of miR-148a associated with more aggressive clinic features

**Fig. 6:**
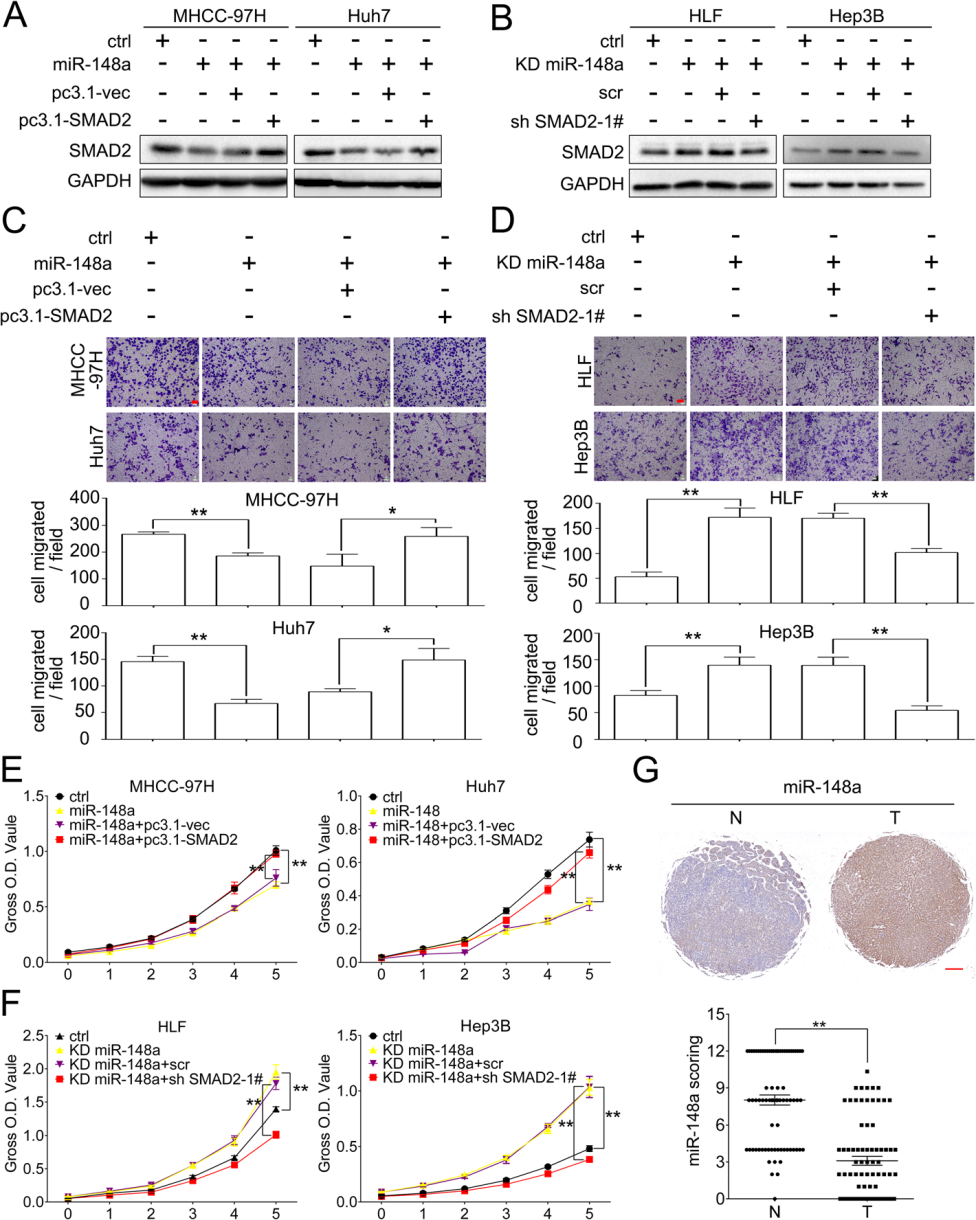
The role of miR-148a in HCC cells was mediated by SMAD2. **a** pcDNA3.1-SMAD2 plasmids were transfected into miR-148a overexpressing MHCC-97H and Huh7 cells to transiently overexpress SMAD2 level. Forty-eight h after transfection, cell lysates were subjected to western blot to evaluate SMAD2 expression. **b** SMAD2 were stably knocked down in miR-148a knocking down HLF and Hep3B cells. Western blot was performed to analyze SMAD2 knocking down efficacy. GAPDH as loading control in (**a**, **b**). **c**, **d** Representative images of migration assay in the indicated cells (**c**, **d**, upper panel). Quantification of cells migrated (**c**, **d**, lower panel). Scale bar, red bar, 25 μm. **e**, **f** CCK8 assay in the indicated cells. **g** Representative ISH images of miR-148a in paired HCC samples (upper panel). Scale bar, red, bar, 200 μm. ISH scoring of miR-148a in 77 pairs of HCC tissues (lower panel). N, non-cancerous tissues. T, tumor tissues. Data are shown as Mean ± SEM. *, *p* < 0.05. **, *p* < 0.01

We further overexpressed SMAD2 in MHCC-97H/miR-148a, Huh7/miR-148a cells using pcDNA3.1-SMAD2 and stably knocked down SMAD2 in HLF/KD miR-148a, Hep3B/KD miR-148a cells (Fig. 6a and b). Transwell and CCK8 assay showed that the impaired migration, invasion and proliferation abilities in MHCC-97H/miR-148a, Huh7/miR-148a cells were recovered by overexpressing SMAD2. In contrast, the enhanced migration, invasion and proliferation abilities in HLF/KD miR-148a, Hep3B/KD miR-148a cells were impeded by knockdown of SMAD2 (Fig. 6c-f).

ISH were performed in 77 pairs of HCC samples (Supplementary Table 2) to investigated the role of miR-148a in clinic. The average miR-148a staining score was significantly lower in HCC samples compared with adjacent non-cancerous tissues (Fig. 6g). *Chi-squared* analysis indicated that lower expression of miR-148a was significantly associated with higher AFP level, HBV infection and higher Child-Pugh score (Supplementary Table 3).

## Discussion

As R-SMAD proteins, the impacts of SMAD2 and SMAD3 on cancer initiation and progression were widely investigated. Interestingly, SMAD2 and SMAD3 were found play distinct, even opposite roles under certain context [10–12]. In non-small-cell lung carcinoma (NSCLC), cancer metastasis was associated with inactivation of SMAD2-mediated and activation of SMAD3-mediated transcriptional programs [31]. During breast cancer bone metastasis progression, the TGF-β induced bone metastatic genes expression were found depend on SMAD3 but not SMAD2, and knockdown of SMAD3 in MDA-MB-231 cells inhibited bone metastasis, while SMAD3 knockdown led to a more aggressive phenotype [8]. In HCC, SMAD3 was reported suppressing carcinogenesis in chemically inducing animal models [32] and sustained SMAD3 activation promoted cancer metastasis [13]. However, the role of SMAD2 in HCC was poorly focused and remained obscure. Our results showed that the expression of SMAD2 was elevated in HCC specimens and high expression of SMAD2 in HCC associated with poor prognosis. SMAD2 promoted proliferation, migration and invasion of HCC cells.

miRNAs can affect TGF-β signaling process by directly targeting canonical members in the signaling pathway or targeting its effector genes [33]. In human, instead of directly slicing targeting mRNA by associated with an Ago protein that retained the catalytic ability of endonucleolytic cleavage, miRNAs mainly partially paired with targeted mRNA, and recruited RISC complexes to enhance mRNA decay or translational repression [17]. In our study, we predicted the mRNA of SMAD2 can be targeted by serials of miRNAs. Among these miRNAs, we identified miR-148a as an upstream regulator of SMAD2 by analyzing the expression relevance between miRNAs and SMAD2, and the association between miRNAs expression and OS in HCC. miR-148a was found downregulated in HCC tissue and low expression of miR-148a was associated with shorter OS time, which is consistent with previous study [21]. We further found that miR-148a downregulating SMAD2 expression through binding with Ago2, the core protein of RISC complexes.

Endeavors have been paid on exploring the effects of miR-148a on the HCC formation and progression. Researchers revealed that miR-148a promoted mouse fetal hepatoblasts (MFHs) differentiating to mature hepatocytes by directly targeting DNA methyltransferase 1 (DNMT1) [23] and induced hepatocytic differentiation to HCC by inhibiting IKKα/NUMB/NOTCH signaling [34]. While, Xiaojie Xu et al. found that Hepatitis B virus X protein (HBx) repressed miRNA-148a to enhance tumorigenesis in mouse animal model of HCC [22]. The role of miRNA-148a as an HCC metastasis repressor seems consistent across different studies, miR-148a-3p suppressed the invasiveness of HCC cell by regulating c-Met [23], Wnt1 [35] or activin A receptor type 1 (ACVR1) [36]. Our results showed that miR-148a inhibited proliferation and migration of HCC cells in vivo and in vitro. And the impacts of miR-148a in HCC cells were mediated by SMAD2.

## Conclusions

This study demonstrated that SMAD2 was highly expressed in HCC specimens, elevated SMAD2 expression was associated with shorter overall survival time for HCC patients. SMAD2 played its tumor promoter role by enhancing migration, invasion and proliferation abilities of HCC cells. Besides, the expression of miR-148a was found negatively related with SMAD2 in HCC. Low expression of miR-148a was associated with more aggressive clinical features and predicted poorer prognosis. miR-148a inhibited HCC cells migration, invasion and proliferation in vitro, suppress tumorigenesis and metastasis in vivo. In mechanism, miR-148a recruited Ago2, bound with and decayed SMAD2 mRNA to inhibit SMAD2 expression. In conclusion, miR-148a was identified as a regulator of oncogenic SMAD2 and may serve as a promising prognostic marker or therapeutic target for HCC patients.

## Supplementary information

**Additional file 1:Supplementary Figure 1.** The expression of SMAD2 and SMAD3 are elevated in HCC tissues compared with counterpart non-tumorous tissues.

**Additional file 2:Supplementary Figure 2.** SMAD3 is upregulated in HCC tissues and high expression of SMAD3 predicted shorter overall survival time.

**Additional file 3:Supplementary Figure 3.** SMAD2 promotes migration, invasion and proliferation of HCC cells.

**Additional file 4:Supplementary Figure 4.** The correlation between the expression of the indicated miRNAs and SMAD2 in HCC patients.

**Additional file 5:Supplementary Figure 5.** miR-148a inhibits metastasis and proliferation of HCC cells in vitro and in vivo.

**Additional file 6:Supplementary Table 1.** The miRNAs co-predicted by three different websites. **Supplementary Table 2.** Clinicopathologic characteristics of patients with hepatocellular carcinoma. **Supplementary Table 3.** Correlation between relative has-miR-148a expression and clinicopathologic characteristics in HCC patients (*n* = 77).

## Data Availability

All data generated or analyzed during this study are included in this published article and its supplementary information files.

## Abbreviations

3’UTR: 3′-Untranslated region
Ago2: Argonaute 2
ACVR1: Activin A receptor type 1
CCK8: Cell counting kit 8
Co-SMAD: Co-mediator SMAD
DNMT1: DNA methyltransferase 1
HBx: Hepatitis B virus X protein
HBV: Hepatitis B virus
HCC: Hepatocellular carcinoma
HCV: Hepatitis C virus
HPIP: Hematopoietic pre–B cell leukemia transcription factor interacting protein
IHC: Immunohistochemistry
ISH: In situ hybridization
I-SMAD: Inhibitory SMAD
LIHC: Liver hepatocellular carcinoma
MFHs: Mouse fetal hepatoblasts,
miRNA: microRNA
miR-148a: miR-148a-3p
miR-27b: miR-27b-3p
NSCLC: Non-small-cell lung carcinoma
OS: Overall survival
PTPRε: Protein tyrosine phosphatase receptor epsilon
qRT-PCR: Quantitative real-time PCR
RIP: Immunoprecipitation
RISC: RNA-induced silencing complex
R-SMAD: Receptor-regulated SMAD
TGF-β: Transforming growth factor-β
