# Supplementary figures and images for: MicroRNA-148a-3p inhibits progression of hepatocelluar carcimoma by repressing SMAD2 expression in an Ago2 dependent manner
Source: J Exp Clin Cancer Res. 2020 Aug 4;39:150. doi: 10.1186/s13046-020-01649-0 (PMC7401232; doi:10.1186/s13046-020-01649-0)

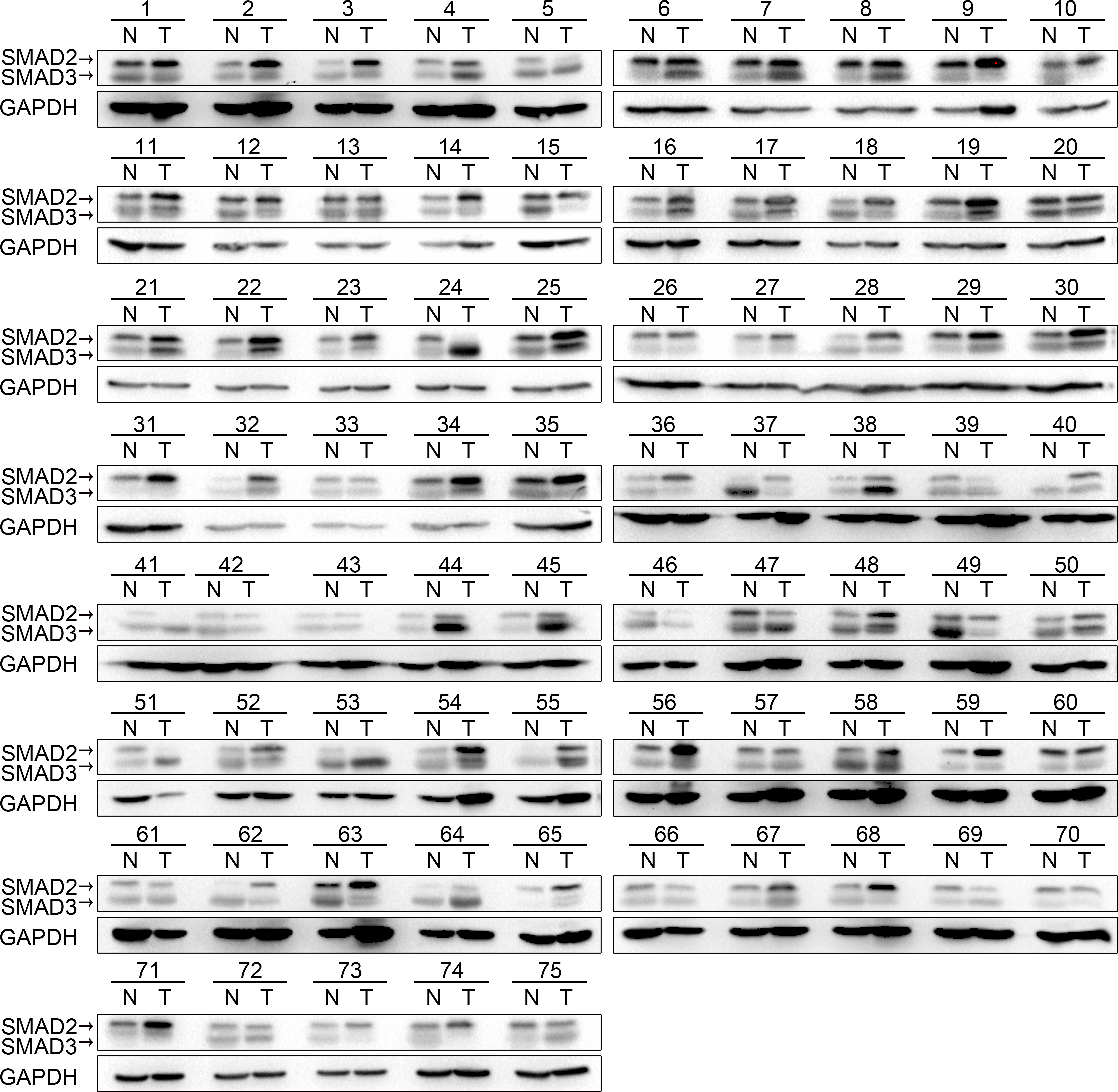

Supplement: Supplementary file 1 — Additional file 1:Supplementary Figure 1. The expression of SMAD2 and SMAD3 are elevated in HCC tissues compared with counterpart non-tumorous tissues. [file 13046_2020_1649_MOESM1_ESM.tif]

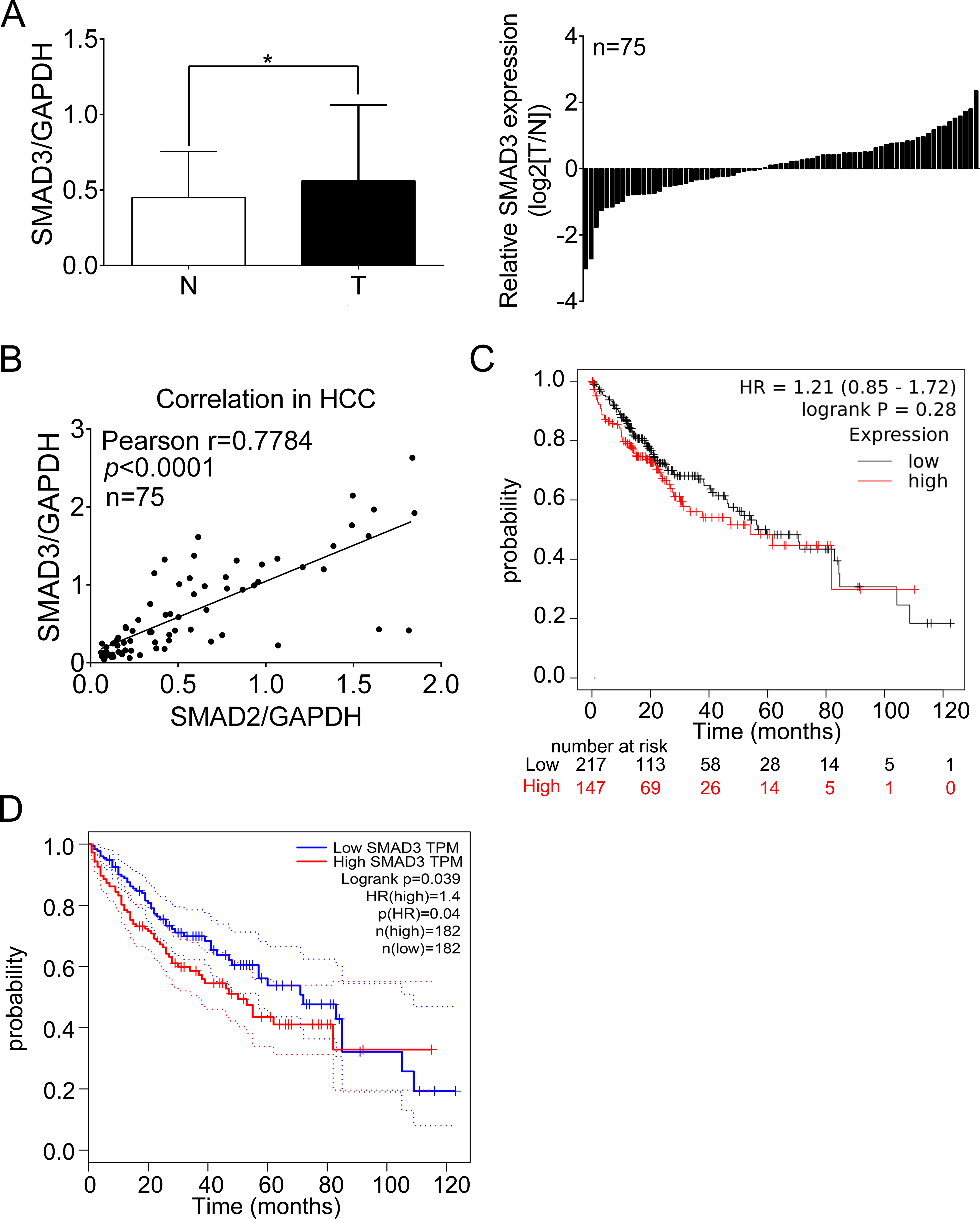

Supplement: Supplementary file 2 — Additional file 2:Supplementary Figure 2. SMAD3 is upregulated in HCC tissues and high expression of SMAD3 predicted shorter overall survival time. [file 13046_2020_1649_MOESM2_ESM.tif]

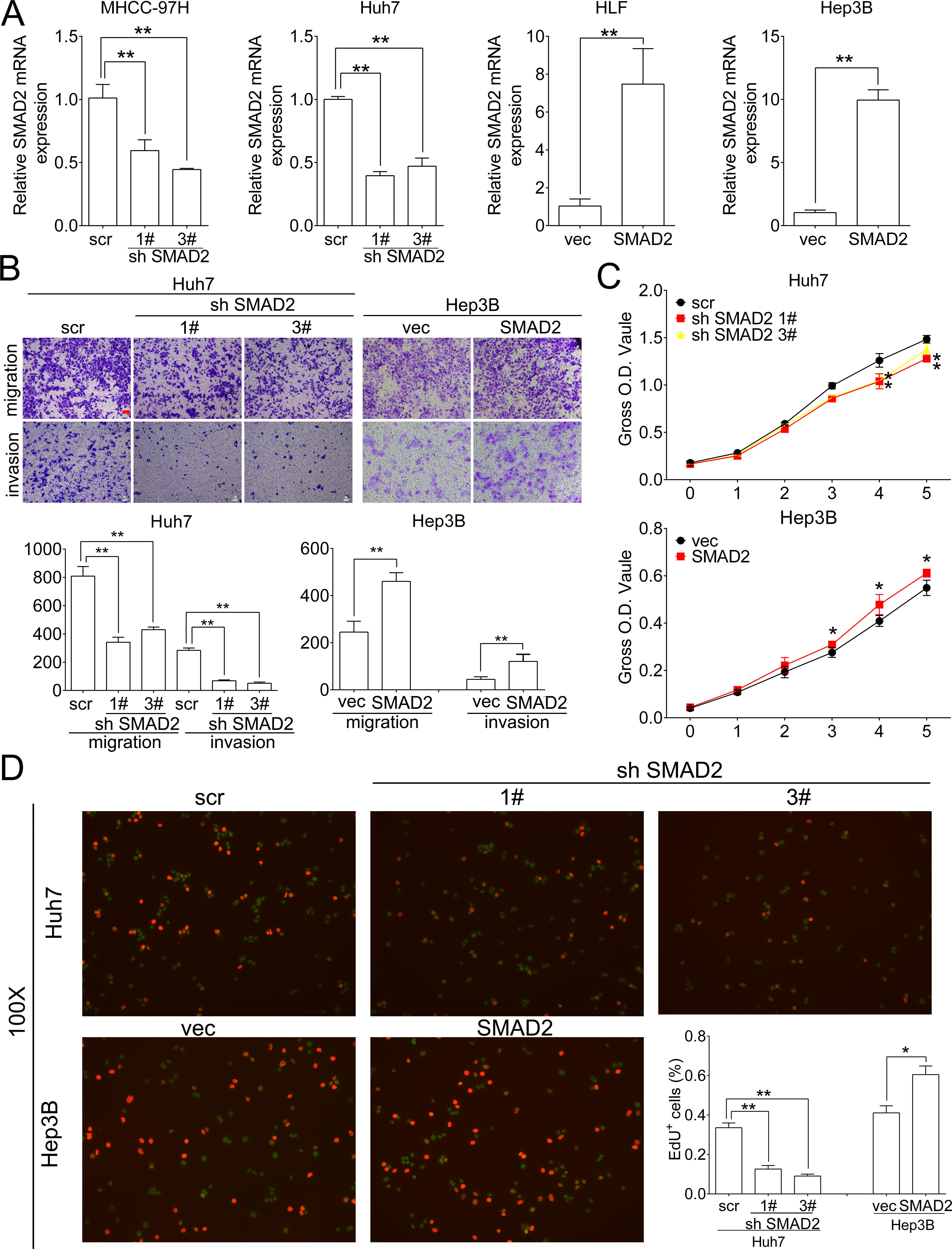

Supplement: Supplementary file 3 — Additional file 3:Supplementary Figure 3. SMAD2 promotes migration, invasion and proliferation of HCC cells. [file 13046_2020_1649_MOESM3_ESM.tif]

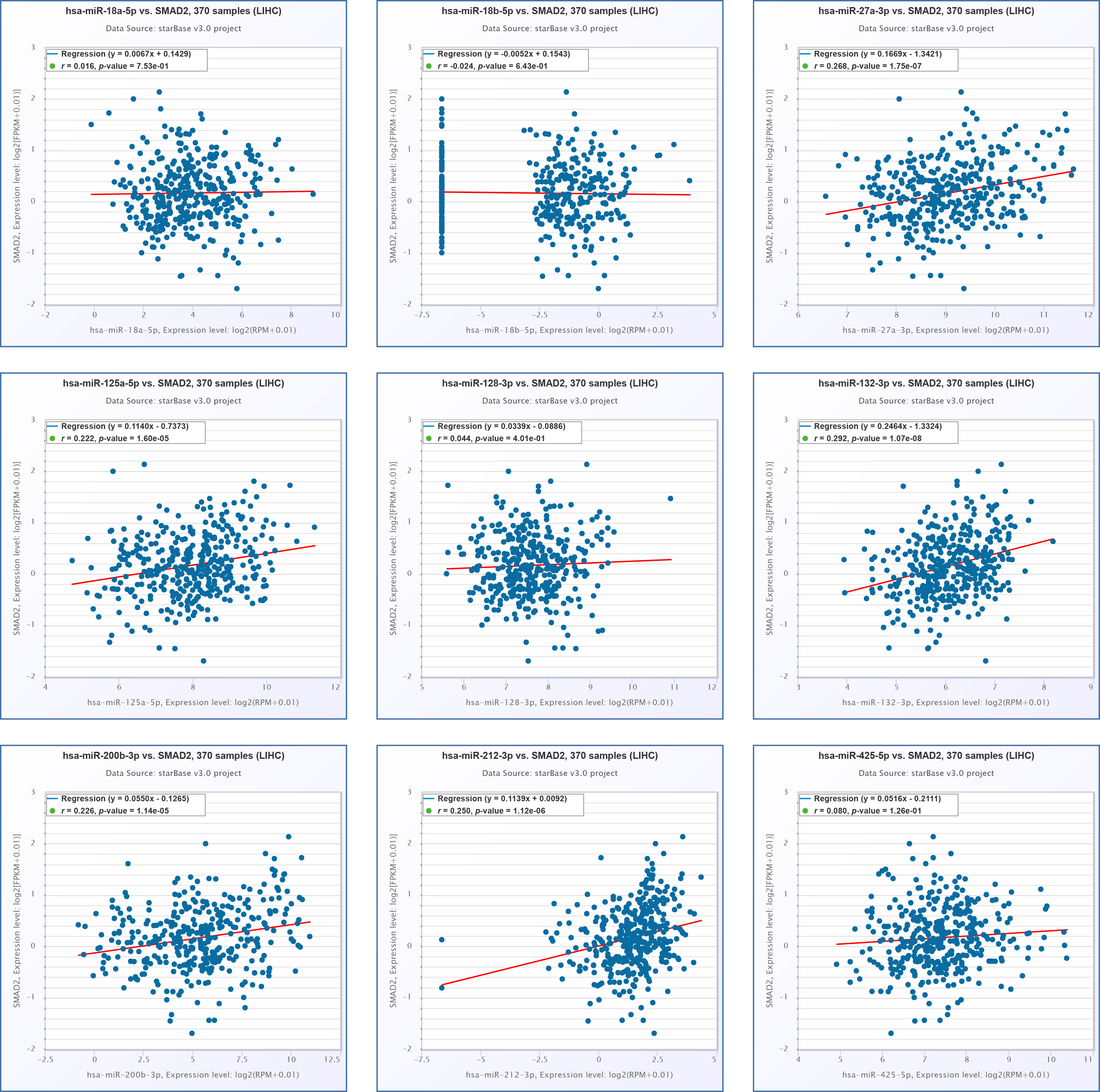

Supplement: Supplementary file 4 — Additional file 4:Supplementary Figure 4. The correlation between the expression of the indicated miRNAs and SMAD2 in HCC patients. [file 13046_2020_1649_MOESM4_ESM.tif]

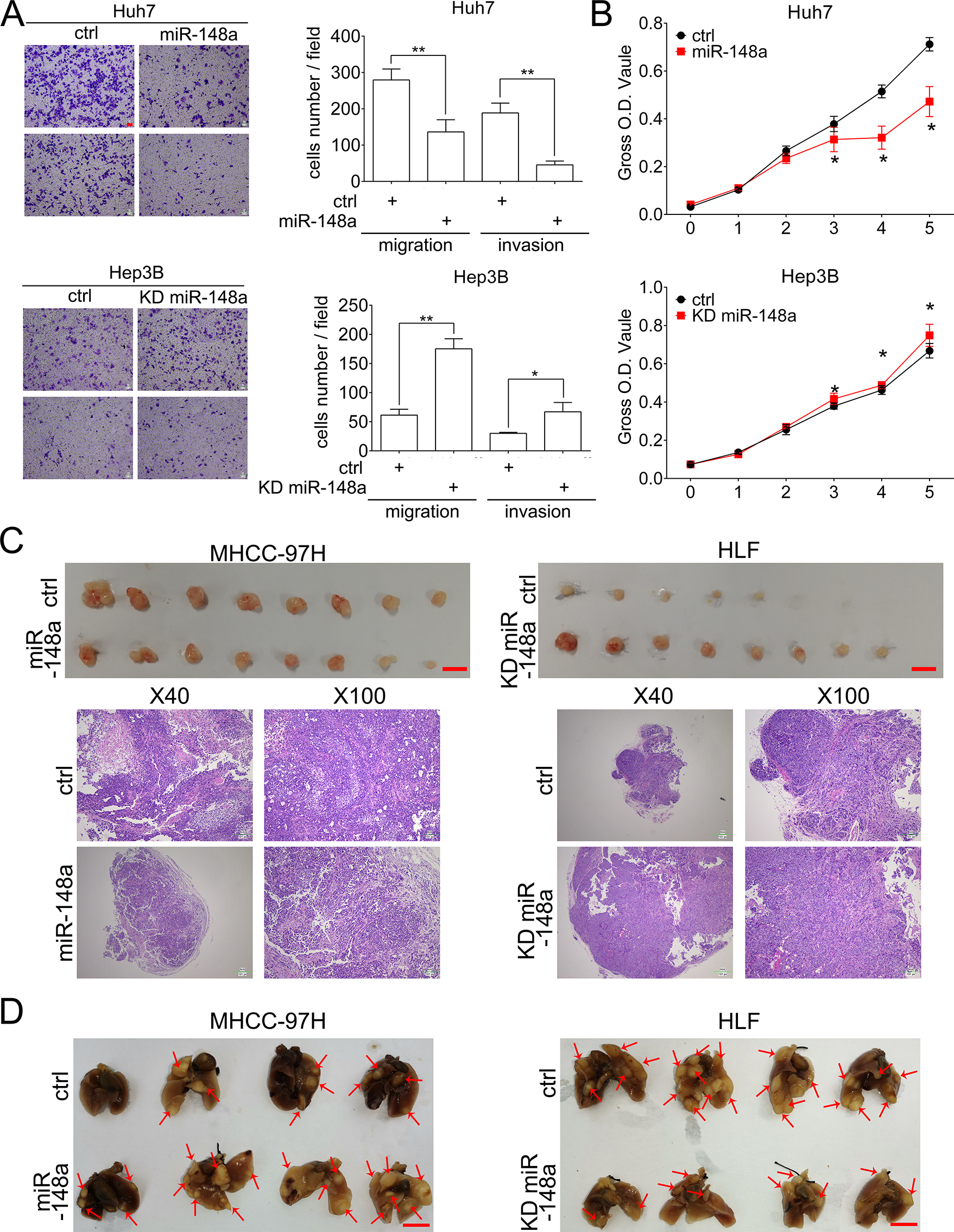

Supplement: Supplementary file 5 — Additional file 5:Supplementary Figure 5. miR-148a inhibits metastasis and proliferation of HCC cells in vitro and in vivo. [file 13046_2020_1649_MOESM5_ESM.tif]
