# Supplementary material for: MicroRNA-148a-3p inhibits progression of hepatocelluar carcimoma by repressing SMAD2 expression in an Ago2 dependent manner
Source: J Exp Clin Cancer Res. 2020 Aug 4;39:150. doi: 10.1186/s13046-020-01649-0 (PMC7401232; doi:10.1186/s13046-020-01649-0)
Supplement: Supplementary file 6 — Additional file 6:Supplementary Table 1. The miRNAs co-predicted by three different websites. Supplementary Table 2. Clinicopathologic characteristics of patients with hepatocellular carcinoma. Supplementary Table 3. Correlation between relative has-miR-148a expression and clinicopathologic characteristics in HCC patients (n = 77). [file 13046_2020_1649_MOESM6_ESM.docx]

**Additional information**

**Supporting materials and methods:**

**Western blot analysis**

Proteins from cultured HCC cells, frozen HCC tissues and normal liver tissues were obtained by RIPA (P0013B, Beyotime, China) supplemented with cOmplete, EDTA-free Protease (04693116001, Roche, Basel, Switzerland) and PhoStop (4906845001, Roche, Basel, Switzerland). Western blot was performed as described previously (1). Primary antibodies included SMAD2 (1:1000, 5339, Cell Signaling Technology), SMAD3 (1:1000, 9523, Cell Signaling Technology), and GAPDH (1:50000, 60004-1-Ig, Proteintech). Optical densities of each protein bands were measured by Image J software (National Institute of Mental Health, USA).

**RNA isolation and the quantitative real-time polymerase chain reaction (qRT-PCR)**

Total RNA was extracted using TRIzol reagent (Invitrogen, Carlsbad, CA, USA) according to the manufacturers’ protocol. We used FastQuant RT Kit (Tiangen, Beijing, China) and miRcute Plus miRNA First-Strand cDNA Synthesis kits (Tiangen, Beijing, China) to reverse transcribe mRNA and miRNA respectively. qRT-PCR was performed using SYBR Green PCR Master Mix-PLUS (TOYOBO, Japan) for mRNA or miRcute Plus miRNA qPCR Detection Kit (Tiangen) for miRNA on ABI ViiA 7 Dx instrument (Applied Biosystems, Foster City, CA, United States) according to manufacturers’ introductions. GAPDH and U6 were used as internal controls for mRNA and miRNA, respectively. We used the comparative CT (2−^∆∆CT^) method to perform relative quantification analysis and each experiment was independently repeated three times at least. Primer sequences: SMAD2-forward, CCGACACACCGAGATCCTAAC, SMAD2-reverse, GAGGTGGCGTTTCTGGAATATAA, GAPDH-forward, CTGGGCTACA -CTGAGCACC, GAPDH-reverse, AAGTGGTCGTTGAGGGCAATG, 3’UTR-SMAD2-forward, TACTGTGCTCCCGTCCAAAC, 3’UTR-SMAD2-reverse, TAGAAAGGTGCAGAGGCAGC. Primers for miRNAs were purchased from RiboBio (Guangzhou, China).

**Plasmid construction and lentivirus package**

pLko.1-TRC plasmid (10879, Addgene, Cambridge, MA) was used as backbone and three short hairpin RNAs (shRNAs) were designed to generate SMAD2 knocking down lentivirus (sh SMAD2 1#, CAAGCACTTGCTCTGA -AATTT, sh SMAD2 2#, ATTGAGCCACAGAGTAATTAT, sh SMAD2 3#, ACTTGAGGTCTCATCAATTAA). Scramble sequence (CCTAAGGTTAAGTCG -CCCTCG) from addgene was used as control. pMD2G, pRRE, pREV were used as packaging plasmids. The complete coding sequence (CDS) of SMAD2 was cloned into pLenti-vector plasmid to generate SMAD2 overexpression lentivirus, pLenti-vector was used as control. pMD2G, pxPAX2 were used as packaging plasmids. The lentivirus packaging procedures were performed as previously described (1).

miR-148a overexpression and knocking down lentivirus were purchased from DesignGene Biotechnology (Shanghai, China).

The CDS of SMAD2 was cloned into pcDNA3.1+ plasmid (Thermo Scientific, Carlsbad, CA, USA) to transient overexpressing SMAD2. pcDNA3.1+/vector was used as control.

**Cell transfection**

We transfected plasmids, miRNA mimics or inhibitors into cells by Lipofectamine 3000 transfection Reagent (Thermo Fisher Scientific, Waltham, MA, United States) following the protocol provided by the manufacturer. miRNA mimics and inhibitors were purchased from RiboBio (Guangzhou, China).

**Transwell assay**

Transwell assays were performed to evaluated cell migration and invasion abilities by 8μm pore size 24-well-transwell plates (Corning, NY, USA) as described previously (1). Three random views were captured under 100x magnification by microscope (Leica, Solms, Germany) for each chamber. The number of staining cells were quantified by Image-Pro Plus v6.0 software.

**Supplementary figure legends:**

**Supplementary figure 1. The expression of SMAD2 and SMAD3 are elevated in HCC tissues compared with counterpart non-tumorous tissues.** Western blot bands of SMAD2 and SMAD3 in 75 pairs HCC tissues. GAPDH as loading control.

**Supplementary figure 2. SMAD3 is upregulated in HCC tissues and high expression of SMAD3 predicted shorter overall survival time.** (A) Quantification of SMAD3 bands intensity in liver tumor and non-tumorous tissues (left panel). Relative SMAD3 expression in HCC tissues by normalizing to their respective adjacent non-cancerous liver tissues (right panel). N, non-tumorous tissues. T, tumor tissues. (B) Correlation analysis between the expression of SMAD2 and SMAD3 in HCC specimens. (C and D) Kaplan-Meier analysis of the correlation between SMAD3 expression and overall survival in HCC patient cohorts by Kaplan Meier-plotter (C) or GEPIA (D) website. Data are shown as Mean ± SEM. **, p<0.01.

**Supplementary figure 3. SMAD2 promotes migration, invasion and proliferation of HCC cells.** (A) qRT-PCR analysis of SMAD2 knocking down or overexpression efficacy in MHCC-97H and Huh7 or HLF and Hep3B cells. Data were normalized to GAPDH and are shown as the fold change to MHCC-97H/scr, Huh7/scr, HLF/vec or Hep3B/vec cells. (B) Representative images of migration and invasion assays after knocking down SMAD2 in Huh7 cells or overexpressing SMAD2 in Hep3B cells (B, upper panel). Scale bar: red bar, 25 µm. Quantification of cells migrated or invaded (B, lower panel). (C, D) CCK8 and EdU assay after knocking down SMAD2 in Huh7 cells or overexpressing SMAD2 in Hep3B cells. Quantification of EdU^+^ cells percentage in (D) (D, bottom panel). Data are shown as Mean ± SEM. *, p<0.05. **, p<0.01.

**Supplementary figure 4. The correlation between the expression of the indicated miRNAs and SMAD2 in HCC patients.** StarBase website was used to evaluate the relevance between SMAD2 mRNA and miR-18a-5p, miR-18b-5p, miR-27a-3p, miR-125a-5p, miR-128-3p, miR-132-3p, miR-200b-3p, miR-212-3p, miR-425-5p in HCC samples. LIHC: liver hepatocellular carcinoma.

**Supplementary figure 5. miR-148a inhibits metastasis and proliferation of HCC cells *in vitro* and *in vivo*.** (A) Representative images of migration and invasion assay in the miR-148a overexpressing Huh7 (Huh7/miR-148a) or miR-148a knocking down HLF (HLF/ KD miR-148a) cells (upper panel). Scale bar: red bar, 25 µm. Quantification of cells migrated and invaded (lower panel). (B) CCK8 assay for the indicated cells. (C) Images of excised subcutaneous tumors from nude mice injected with the indicated cells (upper panel). H&E staining of subcutaneous tumors (lower panel). (D) Excised lungs from nude mice tail-vein injected with the indicated cells. Red arrows, observed metastatic tumor nodules. Scale bar in (C, D), red bar, 1 cm. Data are shown as Mean ± SEM. *, p<0.05. **, p<0.01.

**Supplementary tables:**

Supplementary table 1. The miRNAs co-predicted by three different websites:


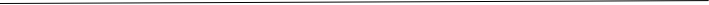


Number miRBase Accession miR name


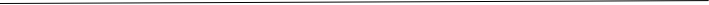


1 MIMAT0000072 hsa-miR-18a-5p

2 MIMAT0001412 hsa-miR-18b-5p

3 MIMAT0000084 hsa-miR-27a-3p

4 MIMAT0000419 hsa-miR-27b-3p

5 MIMAT0000443 hsa-miR-125a-5p

6 MIMAT0000424 hsa-miR-128-3p

7 MIMAT0000426 hsa-miR-132-3p

8 MIMAT0000243 hsa-miR-148a-3p

9 MIMAT0000318 hsa-miR-200b-3p

10 MIMAT0000269 hsa-miR-212-3p

11 MIMAT0003393 hsa-miR-425-5p


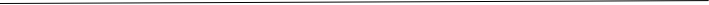


Supplementary table 2. Clinicopathologic characteristics of patients with hepatocellular carcinoma.


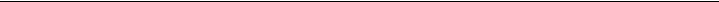


**Clinicopathological Number Percentage**

**variables n=77**


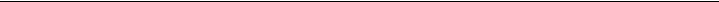


**Gender**

Male 61 0.80

Female 16 0.20

**Age**

≤50 34 0.44

>50 43 0.56

**AFP(ug/L)**

≤20 29 0.37

>20 48 0.62

**GGT(U/L)**

≤54 41 0.53

>54 36 0.47

**HBV**

Negative 15 0.20

Positive 62 0.80

**HCV**

Negative 75 0.97

Positive 2 0.03
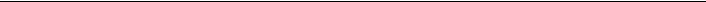


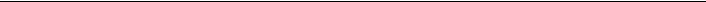


**Cirrhosis**

No 7 0.09

Yes 70 0.91

**Tumor size**

≤5 40 0.52

>5 37 0.48

**Tumor number**

Single 67 0.87

Multiple 10 0.13

**Tumor encapsulation**

None 28 0.36

Complete 49 0.63

**Macrovascular invasion**

No 55 0.71

Yes 22 0.29

**Satellite nodules**

No 61 0.79

Yes 16 0.21

**Child-Pugh**

A 69 0.90

B 8 0.10

**BCLC stage**

0+A 59 0.77

B+C 18 0.23


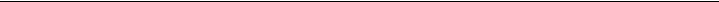


Supplementary table 3. Correlation between relative has-miR-148a expression and clinicopathologic characteristics in HCC patients (n=77).


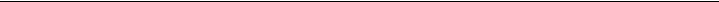


**Clinicopathological Relative expression P value**

**variables Low High**


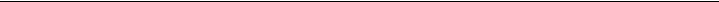


**Gender**

Male 47 14 0.328771816

Female 12 4

**Age**

≤50 30 4 0.318654804

>50 29 14

**AFP(ug/L)**

≤20 20 9 0.008519065*

>20 39 9
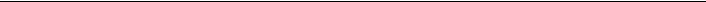

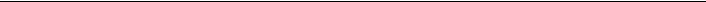


**GGT(U/L)**

≤54 31 10 0.337115768

>55 28 8

**HBV**

Negative 7 8 0.016039063*

Positive 52 10

**HCV**

Negative 58 17 0.215846957

Positive 1 1

**Cirrhosis**

No 4 3 0.051707219

Yes 55 15

**Tumor size**

≤5 31 9 0.168562091

>5 28 9

**Tumor number**

Single 51 16 0.30908474

Multiple 8 2

**Tumor encapsulation**

None 22 6 0.071947189

Complete 37 12

**Macrovascular invasion**

No 42 13 0.176959501

Yes 17 5

**Satellite nodules**

No 47 14 0.309230377

Yes 12 4

**Child-Pugh**

A 52 17 0.035115599*

B 7 1

**BCLC stage**

0+A 43 16 0.260076936

B+C 16 2


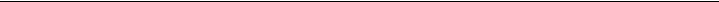
 *represented p value <0.05

1. Ding ZY, Jin GN, Wang W, Chen WX, Wu YH, Ai X, Chen L, et al. Reduced Expression of Transcriptional Intermediary Factor 1 Gamma Promotes Metastasis and Indicates Poor Prognosis of Hepatocellular Carcinoma. Hepatology 2014;60:1620-1636.
